# ADH1B promotes mesothelial clearance and ovarian cancer infiltration

**DOI:** 10.18632/oncotarget.25344

**Published:** 2018-05-18

**Authors:** Kshipra M. Gharpure, Olivia D. Lara, Yunfei Wen, Sunila Pradeep, Chris LaFargue, Cristina Ivan, Rajesha Rupaimoole, Wei Hu, Lingegowda S. Mangala, Sherry Y. Wu, Archana S. Nagaraja, Keith Baggerly, Anil K. Sood

**Affiliations:** ^1^ Department of Gynecologic Oncology and Reproductive Medicine, The University of Texas MD Anderson Cancer Center, Houston, TX 77030, USA; ^2^ Department of Experimental Therapeutics, The University of Texas MD Anderson Cancer Center, Houston, TX 77030, USA; ^3^ Center for RNA Interference and Non-Coding RNA, The University of Texas MD Anderson Cancer Center, Houston, TX 77030, USA; ^4^ Department of Pathology, Institute of RNA Medicine, Beth Israel Deaconess Medical Center Cancer Center, Harvard Medical School, Boston, MA 02215, USA; ^5^ Department of Bioinformatics and Computational Biology, The University of Texas MD Anderson Cancer Center, Houston, TX 77030, USA; ^6^ Department of Cancer Biology, The University of Texas MD Anderson Cancer Center, Houston, TX 77030, USA

**Keywords:** alcohol dehydrogenase, residual disease, mesothelial clearance, ECM degradation

## Abstract

Primary debulking surgery followed by adjuvant chemotherapy is the standard treatment for ovarian cancer. Residual disease after primary surgery is associated with poor patient outcome. Previously, we discovered ADH1B to be a molecular biomarker of residual disease. In the current study, we investigated the functional role of ADH1B in promoting ovarian cancer cell invasiveness and contributing to residual disease. We discovered that ADH1B overexpression leads to a more infiltrative cancer cell phenotype, promotes metastasis, increases the adhesion of cancer cells to mesothelial cells, and increases extracellular matrix degradation. Live cell imaging revealed that ADH1B-overexpressing cancer cells efficiently cleared the mesothelial cell layer compared to control cells. Moreover, gene array analysis revealed that ADH1B affects several pathways related to the migration and invasion of cancer cells. We also discovered that hypoxia increases ADH1B expression in ovarian cancer cells. Collectively, these findings indicate that ADH1B plays an important role in the pathways that promote ovarian cancer cell infiltration and may increase the likelihood of residual disease following surgery.

## INTRODUCTION

Primary debulking surgery and adjuvant chemotherapy are the standard treatment for ovarian cancer. After primary surgery the remaining tumor is referred to as residual disease. Residual disease following primary surgery is associated with poor overall and progression-free survival [1, 2]. Residual disease may be attributed to a lack of sufficient surgical skill, but recent studies suggest that tumor biology also plays an important role in the persistence of ovarian cancer after surgery [2, 3]. Although researchers are working to develop biomarkers that can predict the likelihood of residual disease following primary surgery [3–5], the specifics of tumor biology responsible for residual ovarian cancer remain unclear.

In our previous study, we discovered that alcohol dehydrogenase 1B (ADH1B) is a promising molecular biomarker for predicting residual ovarian cancer [3]. The physiological function of ADH1B has mainly been studied in the context of ethanol metabolism. ADH1B metabolizes ethanol to acetaldehyde, which is further oxidized to acetic acid by aldehyde dehydrogenase. Acetaldehyde, if accumulated beyond a certain threshold, can intercalate with DNA to form carcinogenic adducts that increase the chances of cancer initiation. Acetaldehyde also affects the DNA repair machinery; it inhibits O6 methyl-guanyltransferase and thus can cause oncogenic mutations [6, 7]. ADH1B mutations have been extensively studied and have been linked to various types of cancer. The functional polymorphism ADH1B rs1229984 (Arg47His) has been associated with esophageal, head and neck, and colorectal cancers [8–10]. However, the biological roles of ADH1B in metastasis and residual disease are unknown. The discovery of high ADH1B expression in those with residual disease in our previous study prompted us to further investigate whether ADH1B plays a functional role in this process.

Residual disease may, in part, be related to the more infiltrative nature of some ovarian cancers. All peritoneal organs are covered with a layer of mesothelial cells. During progression, ovarian cancer cells detach from the primary tumor site, form spheroids, and then adhere to and invade the peritoneal organs [11]. Studies using electron microscopy have shown that the mesothelial cell layer surrounding healthy peritoneal organs is intact, whereas that surrounding peritoneal organs with metastases has gaps, indicating that invading cancer cells caused the mesothelial cells to retract [12, 13].

The purpose of the present study was to determine the extent to which ADH1B has a functional role in ovarian cancer invasiveness, which may contribute to increased likelihood of leaving residual disease.

## RESULTS

### ADH1B promotes tumor progression

**Figure 1:**
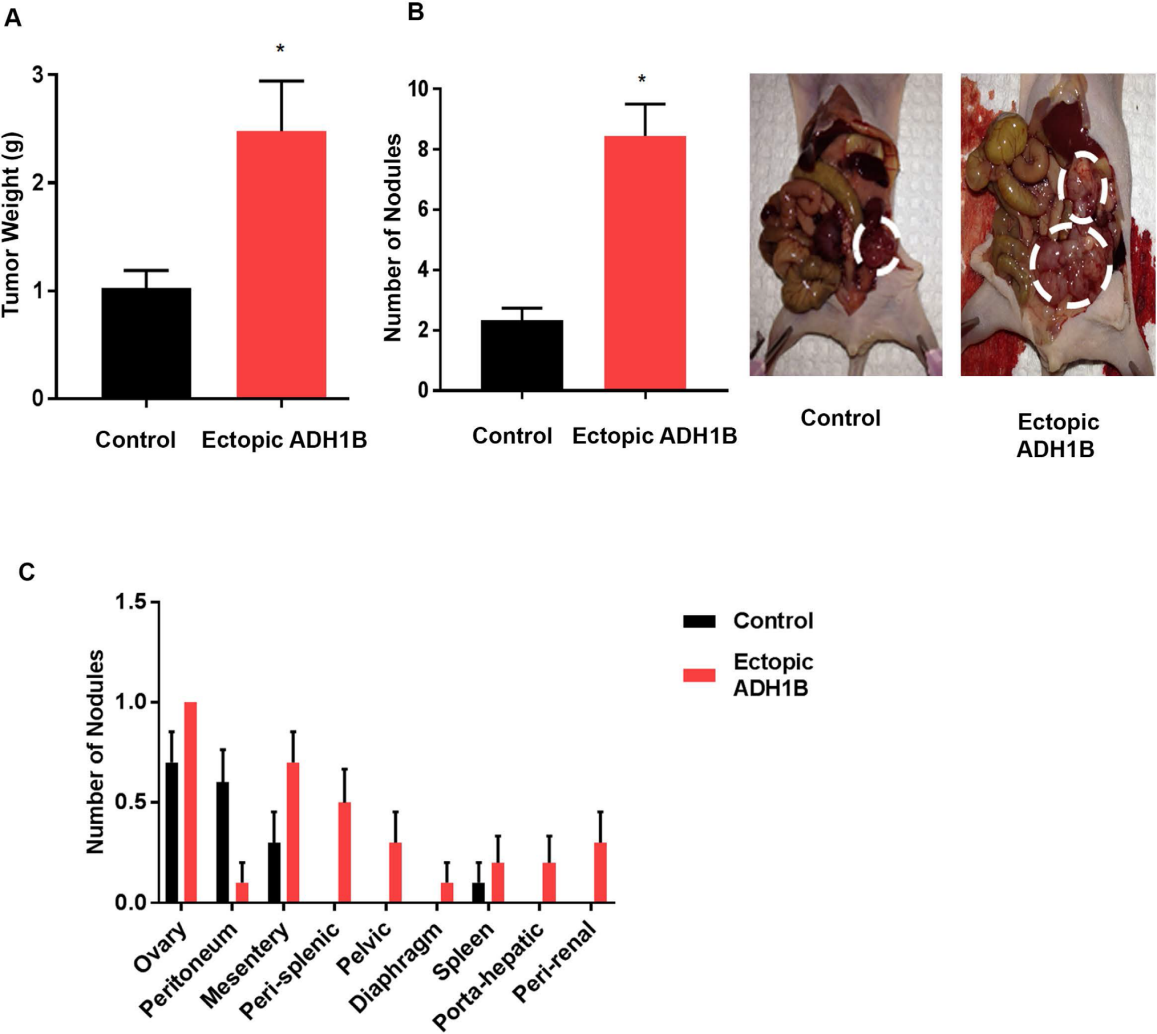
ADH1B promotes tumor progression (**A**) Aggregate masses of tumors from orthotopic mouse models injected with A2780cells transfected with an ADH1B-expressing vector or control vector (*n* = 10 mice per group). ^*^p < 0.01 is for tumor weight in A2870cells vs. control. (**B**) Representative images show the numbers of nodules in mice injected with ADH1B-overexpressing cells or control cells (*n* = 10 mice per group). ^*^p < 0.01 is number of nodules in ADH1B overexpressing cells vs. control. (**C**) Distribution of tumor nodules in the mice models. Data for all figures are represented as mean ± SEM.

Having established the role of ADH1B as a molecular predictor of residual ovarian cancer [3], we sought to determine the functional role of ADH1B in ovarian cancer metastasis. We first established cell lines with stable ectopic ADH1B expression by transfecting A2780 cells with either an ADH1B-overexpressing or a control lentivirus. We injected both types of cells directly into the ovaries of nude mice through a laparotomy. Mice were sacrificed when any mouse became moribund. At the end of the experiment, the aggregate tumor weight of the mice injected with cells with ectopic ADH1B expression was significantly higher (~2.5-fold) than those of the mice injected with control cells (*p* < 0.01, Figure 1A). The mice injected with cells with ectopic ADH1B expression also had significantly more nodules than the mice injected with the control cells did (*p* < 0.01, Figure 1B). The mice injected with the ADH1B overexpressing cells also had higher rates of metastases at mesenteric, pelvic, diaphragmatic, renal, hepatic, and splenic sites, whereas the mice injected with the control cells had tumors mainly at the site of injection (i.e., the ovary, Figure 1C). Immunohistochemical analysis confirmed that tumors from mice injected with cells with ectopic ADH1B expression had higher ADH1B expression than mice injected with control cells did (Supplementary Figure 1).

### ADH1B enhances mesothelial clearance in ovarian cancer

**Figure 2:**
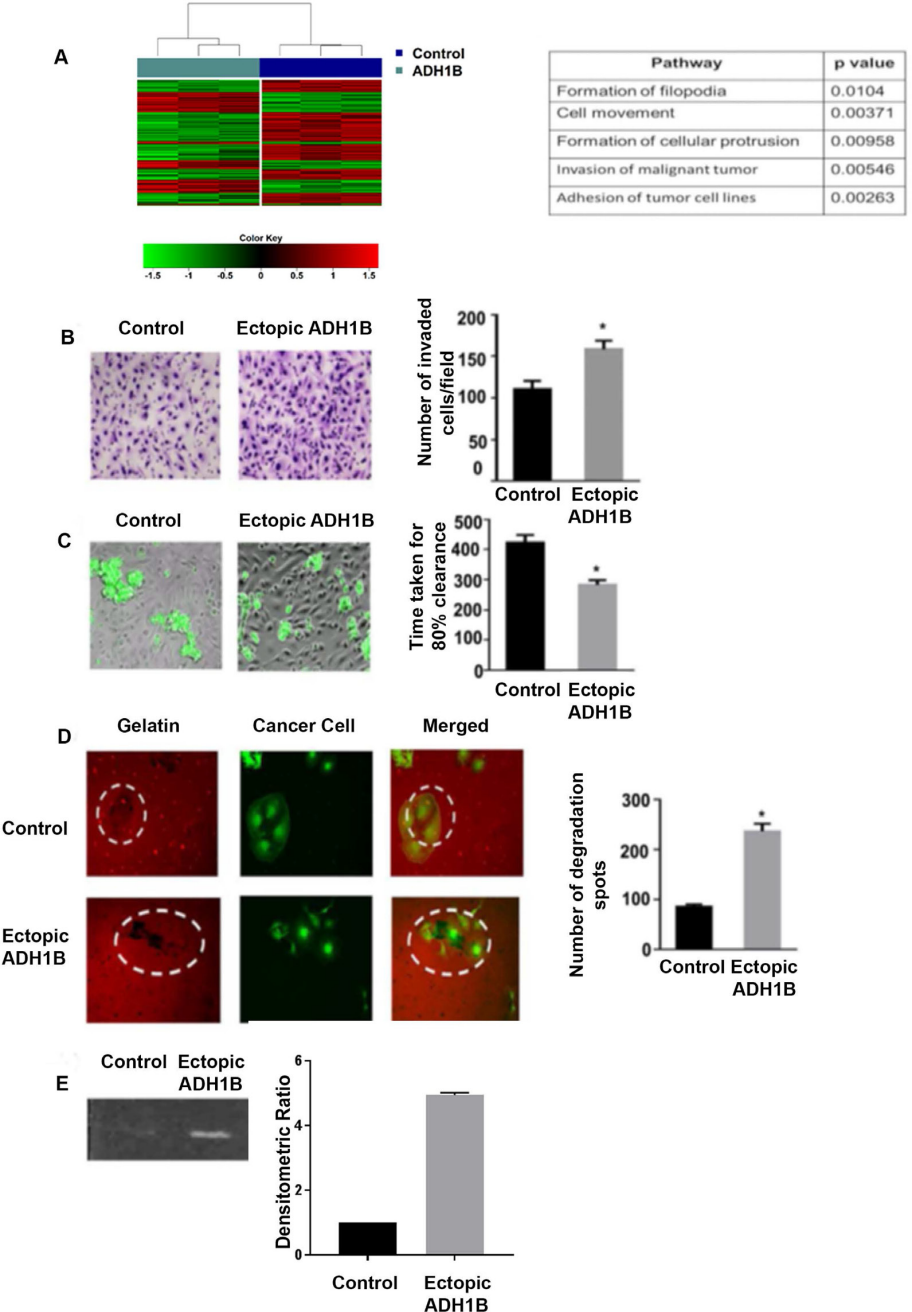
ADH1B enhances mesothelial clearance in ovarian cancer (**A**) Ingenuity Pathway Analysis identified pathways that are significantly altered after ADH1B upregulation. Hierarchical clustering and heatmap using the 300 most variant probes across the samples from the two groups: Control and ADH1B. Red indicates elevated expression, green reduced expression. (**B**) Effect of ADH1B upregulation on SKOV3ip1 cell invasion through the mesothelial cell layer. ^*^p < 0.05 is for number of invaded cells ectopic ADH1B cells vs. control. (**C**) Representative images of live cell imaging performed to determine the mesothelial clearance of spheroids of ADH1B-overexpressing cells and control cells. ^*^p < 0.05 is for spheroids of ADH1B-overexpressing cells that had faster mesothelial clearance than spheroids of control cells. (**D**) Gelatin degradation assay was performed using conditioned media from cells with ectopic ADH1B expression or from control cells.^*^p < 0.05 is for ability of ADH1B overexpressing cells vs control cells to achieve gelatin degradation. (**E**) Zymography was performed with conditioned media from cells ectopically expressing ADH1B or from control cells. Conditioned media from ADH1B overexpressing cells achieved more degradation (~2.5 fold) than the conditioned media from control cells. Data for all figures are represented as mean ± SEM.

We next conducted a gene array analysis of cells with ectopic ADH1B expression and control cells. Ingenuity Pathway Analysis identified many metastasis-related pathways, including those underlying filopodia formation, cell movement, cellular protrusion formation, malignant tumor invasion, tumor cell adhesion, and cell spreading were significantly upregulated in ADH1B-overexpressing cells compared with control cells (Figure 2A).

To invade the peritoneal cavity, ovarian cancer cells must first infiltrate into the mesothelial monolayer. Because we observed that cells ectopically expressing ADH1B are more invasive *in vivo* than control cells, we next examined whether cells ectopically expressing ADH1B pass through a layer of mesothelial cells more quickly than control cells do. An invasion assay revealed that after 24 hours, 65 percent more cells with ADH1B expression passed through the layer when compared to control cells, (*p* < 0.05, Figure 2B). We performed live cell imaging to further investigate the invasion of ADH1B-overexpressing cells. First, we plated spheroids of ADH1B-overexpressing cells or spheroids of control cancer cells on the mesothelial layer and assessed the time to achieve 80% clearance of mesothelial cells for each group. ADH1B-overexpressing spheroids achieved 80% clearance faster than the control spheroids did (*p* < 0.05, Figure 2C). Next, we treated the mesothelial cells with conditioned media from ADH1B-overexpressing or from control cells and then used spheroids of control cells in both the groups. Live cell imaging revealed that spheroids on mesothelial cells treated with conditioned media from ADH1B-overexpressing cells achieved 80% clearance faster than those on mesothelial cells treated with media from control cells did (Supplementary Figure 2A).

Before cancer cells can invade a mesothelial cell layer, they need to adhere to this layer. Hence, we tested whether ADH1B-overexpressing cells or control cells have better adherence to mesothelial cells. First, we investigated whether ectopic ADH1B expression results in increased expression of any genes that may play a role in this process. We analyzed control and ADH1B overexpressing cells with qRT-PCR for the expression of such genes and found that ADH1B overexpression increases CD44 and CD47 expression in the cancer cells (*p* < 0.001 and *p* < 0.001, Supplementary Figure 2B). We next counted the ADH1B-overexpressing cells or control cells adhering to the mesothelial monolayer at specific times and found that more ADH1B-overexpressing cells than control cells adhered to the mesothelial cells (*p* < 0.05, Supplementary Figure 2C).

Next, we performed a gelatin degradation assay to assess the ability of ADH1B-overexpressing cells to degrade extracellular matrix. We conducted a gelatin degradation assay with the conditioned media collected from control or ADH1B overexpressing cells. We plated control or ADH1B overexpressing cells on layers of Cy3-gelatin and counted the degradation spots for both groups. ADH1B-overexpressing spheroids led to significantly more gelatin degradation than control cells did (*p* < 0.05, Figure 2D). To obtain quantitative data, we conducted zymography using conditioned media from ADH1B-ovexpressing cells or from control cells. Again, the conditioned media from ADH1B-overexpressing cells degraded more (~2.5 fold) gelatin than that conditioned with control cells (Figure 2E), suggesting that ADH1B-overexpressing cells have a greater ability to degrade extracellular matrix and thus invade mesothelial cells than do cells without ADH1B expression. Next, we conducted a protease proteome profiler assay using conditioned media from ADH1B-overexpressing cells or from control cells. We found that cathepsin V, MMP-7, and CD26 are substantially upregulated (~2.0 fold) in conditioned media from ADH1B-overexpressing cells (Supplementary Figure 2D).

### Regulation of ADH1B expression in ovarian cancer cells

**Figure 3:**
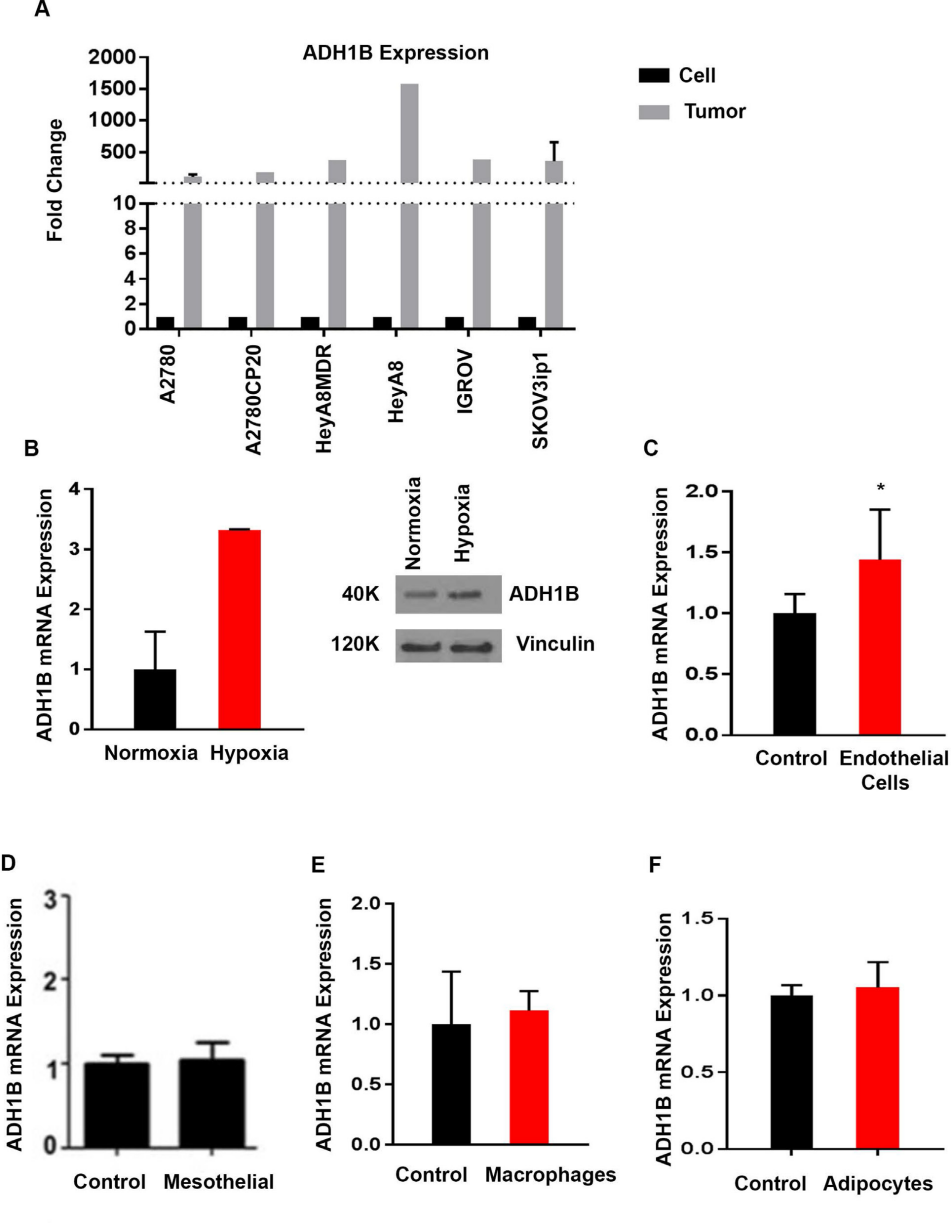
Regulation of ADH1B in ovarian cancer cells (**A**) ADH1B expression in cancer cells *versus* that in tumor tissue from mice injected with the corresponding cell line. (**B**) Effect of hypoxia on mRNA expression of ADH1B in SKOV3ip1 cancer cells ^*^p < 0.05 is for ADH1B expression in hypoxic cells vs. normal conditions. ADH1B protein overexpression validated by western blot. (**C**) Effect of the conditioned media from endothelial cells (RF24) on the expression of ADH1B. ^*^p < 0.05 is for ADH1B expression in conditioned endothelial cells of RF24 v control. (**D**) Effect of the conditioned media from mesothelial cells on ADH1B expression. (**E**) Effect of the conditioned media from macrophages (differentiated THP1 cells) on ADH1B expression. (**F**) Effect of the conditioned media from adipocytes on ADH1B expression. Data for all figures are represented as mean ± SEM.

Next, we investigated the upstream mechanism that regulates ADH1B expression in ovarian cancer cells. We first assessed the extent to which copy number and ADH1B mRNA expression are related in ovarian cancer patient samples. We analyzed The Cancer Genome Atlas (TCGA) data obtained with Affymetrix and Agilent platforms for patients with high-grade serous ovarian cancer and found no association between copy number and ADH1B expression (Supplementary Figure 3A). We then assessed the expression of ADH1B in ovarian cancer cell lines. All cell lines had fairly low ADH1B expression (Supplementary Figure 3B), which prompted us to compare the ADH1B expression in tumor samples from mice injected with a cell line with the ADH1B expression in the same cell line *in vitro*. ADH1B expression was significantly higher in the tumor samples than in the cell lines (Figure 3A), suggesting that tumor microenvironmental factors increase ADH1B expression *in vivo*.

We then investigated the effects of various factors on ADH1B expression in cancer cells. We treated SKOV3ip1 ovarian cancer cells with conditioned media from mesothelial cells, endothelial cells, macrophages, or fibroblasts and assessed their ADH1B expression. We also assessed the effects of a hypoxic environment (1% oxygen) on the cells’ ADH1B expression. The level of ADH1B expression in cells conditioned in hypoxic conditions was approximately 3 times that of control cells (*p* < 0.05, Figure 3B). The effect was also observed in HeyA8 MDR cell line, (Supplementary Figure 3C). We assessed the cells’ carbonic anhydrase IX levels to ensure that the cells were truly hypoxic. The conditioned media from endothelial cells significantly increased ADH1B expression in two cell lines, RF24 (*p* < 0.05, Figure 3C) and HEYA-8 MDR (*p* < 0.05, Supplementary Figure 3D), whereas conditioned media from macrophages, mesothelial cells, or adipocytes had no effect on ADH1B expression (Figure 3D–3F and Supplementary Figure 3E). The most significant and consistent effect was observed under hypoxic conditions.

### ADH1B is associated with poor survival

**Figure 4:**
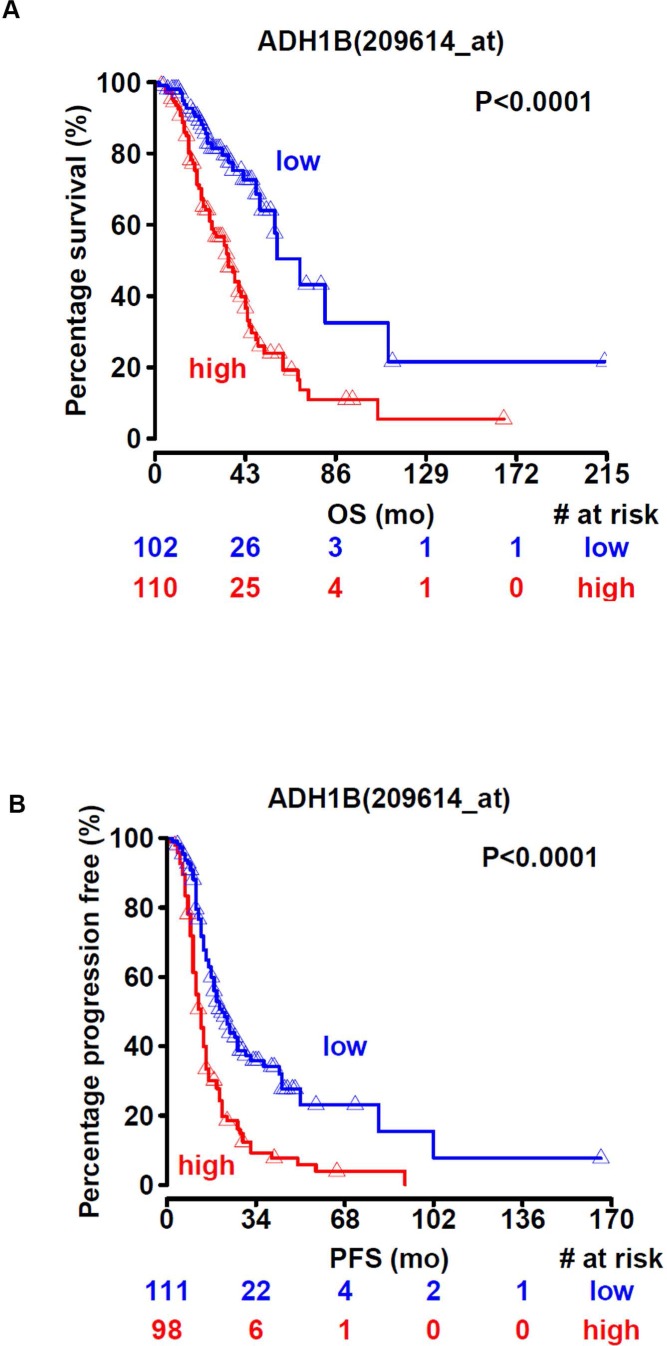
ADH1B is associated with poor survival (**A** and **B**) High ADH1B expression is associated with lower overall survival (A) and progression-free survival (B) in ovarian cancer patients.

We analyzed publicly available datasets of ovarian cancer patients to determine the effect of ADH1B expression on survival. Specifically, we focused on high-grade and advanced-stage ovarian cancer cases. Increased ADH1B expression was associated with worse overall (*p* < 0.0001, Figure 4A) and worse progression-free survival (*p* < 0.0001, Figure 4B).

## DISCUSSION

The findings of our study establish a link between ADH1B and a more infiltrative phenotype. Our preclinical data show that ADH1B upregulation promotes tumor progression and cancer cell invasiveness. ADH1B-overexpressing cells achieve mesothelial clearance faster than other cancer cells do and thus can easily infiltrate the peritoneal cavity. The results of our live cell imaging studies demonstrate the dynamic process of cancer cells’ clearance of the mesothelial cell layer and the importance of ADH1B in this process. Zymography and gelatin degradation assays provide further evidence that ADH1B upregulation helps cells secrete proteases to degrade extracellular matrix. Investigating the upstream regulation of ADH1B, we examined several possibilities that can regulate gene expression under *in vivo* conditions and narrowed down the effect to hypoxic tumor micro-environment. Hypoxia has been previously associated with tumor progression in ovarian cancer. However, regulation of ADH1B by hypoxia was not known.

Iwanicki *et al.* first showed that ovarian cancer cells use integrin and actinomycin contractility to exert force on fibronectin in the mesothelial monolayer, thus achieving mesothelial clearance [14]. The same group also profiled mesothelial clearance–competent and –incompetent ovarian cancer cell lines and concluded that mesenchymal genes, including *SNAI1*, *ZEB1*, and *TWIST1* are enriched in the clearance-competent cells [15]. Kenny *et al.* also studied the interaction between ovarian cancer cells and mesothelial cells. Their study showed that TGF-β1 secreted by cancer cells activates the TGF-β receptor/RAC1/SMAD–dependent signaling pathway in mesothelial cells. The mesothelial cells then undergo epithelial-to-mesenchymal transformation and secrete fibronectin, which in turn increases the adhesion, invasion, proliferation, and metastasis of ovarian cancer cells [16]. The current study establishes that ADH1B also plays a crucial role in ovarian cancer cells achieving mesothelial clearance.

Our study's findings demonstrate that ADH1B promotes several factors that further contribute to ovarian cancer progression. We found that cells with ADH1B expression secrete MMP-7, CD-26, and cathepsins, which promote cancer progression by their action as proteases. MMP-7, which is overexpressed in ovarian cancer cell lines as well as in patient tumor tissue specimens, also promotes invasion through pro-gelatinase activation [17, 18]. CD-26 regulates several cytokines and chemokines and is overexpressed in ovarian cancer [19, 20]. Cathepsins have diverse roles in cancer biology and cathepsin V promotes cancer cell invasion [21, 22]. In addition, CD44 and CD47, which we found to be upregulated after ADH1B upregulation, help in metastasis by promoting cancer cells’ adhesion to peritoneum [23–27]. Our zymography studies indicated that conditioned media from ADH1B-overexpressing cells contains MMP-2 and MMP-9, which have long been associated with increased degradation of extracellular matrix; in addition, increased MMP-2 and MMP-9 expression levels have been linked with aggressive phenotypes and increased metastasis [28–31].

In summary, the findings of the present study demonstrate that ADH1B upregulation plays an important role in determining the infiltrative phenotype of ovarian cancer cells. We discovered a novel mechanism by which ovarian cancer cells with ADH1B overexpression become more infiltrative and can more efficiently penetrate the mesothelial cell monolayer. The findings of this study shed light on the underlying biology of residual disease and establish ADH1B as a potential target for future therapeutic interventions.

## MATERIALS AND METHODS

### Cell line maintenance

All cell lines were maintained at 37° C in a 5% CO_2_ atmosphere. Cells were cultured in RPMI-1640 supplemented with 15% fetal bovine serum (FBS) and 0.1% gentamycin. RF-24 cells were maintained in MEM supplemented with 10% FBS, pyruvate, amino acids, penicillin and streptomycin. Mesothelial cells were maintained in RPMI 1640 medium supplemented with 10% FBS, amino acids and penicillin/streptomycin. Cells at 60–80% confluence were used for all *in vitro* experiments. To obtain cells with ectopic ADH1B expression, we transfected the cells with a lentivirus containing ADH1B and green fluorescence protein (GFP). The cells were then selected for GFP using flow cytometry. All the selected cells thus ectopically expressed ADH1B. The control cells also received a similar transfection with a lentivirus containing only a GFP-expressing plasmid. The cells then underwent a similar selection process.

### *In vivo* experiments

Female athymic nude mice were purchased from Taconic Farms (Hudson, NY) and housed in pathogen-free conditions. The protocol for the *in vivo* experiment was approved by MD Anderson's Institutional Animal Care and Use Committee. The mice were cared for according to the guidelines of the American Association for Accreditation for Laboratory Animal Care International and the U.S. Public Health Service Policy on Humane Care and Use of Laboratory Animals.

For the *in vivo* experiment, the mice were divided into two groups; one received control cells, and the other received cells ectopically expressing ADH1B. There were 10 mice in each group. Mice were injected with 1 × 10^6^ cells in the left ovary. The mice received no therapeutic intervention and were regularly monitored. When a mouse from either group became moribund, all mice were euthanized. Each mouse's weight, tumor weight, nodule numbers, and metastasis locations were recorded. Excised tumor tissues were then either snap-frozen fixed in formalin for paraffin embedding or frozen in optimum cutting temperature media.

### Invasion assay

8 micron PET membrane cell culture inserts were coated with defined basement membrane matrix prepared in a 10-ml stock solution with laminin (50 μg/ml)-1 ml, type IV collagen (50 μg/ml)- 0.2 ml and gelatin (2 mg/ml)-4 ml and 4.8 ml of PBS. The mesothelial cells were plated on this matrix to form a monolayer. SKOV3ip1 cells ectopically expressing ADH1B or control cells were then suspended in 200 μl of serum-free media and were added to the upper chamber. In the lower chamber, 500 μl of complete media with 15% FBS was added as a chemo-attractant. The cells were then incubated at 37° C and in a 5% CO_2_ atmosphere. After 24 hours, cells in the upper chamber were removed, fixed, and stained and then counted using light microscopy. Five random fields were used for counting.

### Immunoblotting

Lysates from cultured cells were prepared using modified RIPA buffer (50 mM Tris–HCl (pH 7.4), 150 mM NaCl, 1% Triton, 0.5% deoxycholate). The protein concentrations were determined using a BCA Protein Assay Reagent kit (Pierce Biotechnology, Rockford, IL). Lysates were loaded and separated on SDS–PAGE. Proteins were transferred to a nitrocellulose membrane by wet electrophoresis (Bio-Rad Laboratories, Hercules, CA) overnight, blocked with 5% BSA for one hour and then incubated at 4° C overnight with primary antibody (ADH 1:1000, Cell signaling, Danvers, MA). After washing with tris-buffered saline with 1% Tween 20, membranes were then incubated with horseradish peroxidase-conjugated horse anti- Rabbit IgG (1:2000, GE Healthcare, UK) for two hours. Visualization of horseradish peroxidase was performed using an enhanced chemiluminescence detection kit (Pierce Biotechnology). To confirm equal sample loading, the blots were probed with an antibody specific for vinculin (1:3000 dilution; Sigma-Aldrich, St. Louis, MO).

### Mesothelial cells clearance assay

A 96 well plate was used for this assay. Fibronectin (Sigma, F1141) was used to coat the wells. Mesothelial cells were then plated to form a monolayer on the layer of fibronectin. Control or ADH1B overexpressing cells (SKOV3ip1-GFP) were plated on the mesothelial cells. The invasion of GFP SKOV3ip1 cells were visualized with a laser scanning multiphoton confocal microscope (TCS SP5 MP; Leica Microsystems, Buffalo Grove, IL) for time course analysis. Images of 20+ spheroid/monolayer interactions were collected, every 10 minutes, for up to 12 hours. The GFP-expressing SKOV3ip1 spheroids invaded into the mesothelial cell monolayer creating a hole in the monolayer, and the time taken for completing 80% clearance had been compared statistically between control and ectopic ADH1B expressing cells. Each experiment was repeated at least three times, and representative images were identified and combined in Image J software to get invasion video.

### Gelatin degradation assay

To assess gelatin degradation, we conducted a QCM™ Gelatin Invadopodia Assay (cat. #ECM671, Millipore, Billerica, MA) according to the manufacturer's instructions. Briefly, the 8-well chamber slides were coated with poly-L-lysine and then glutaraldehyde. The wells were then coated with Cy3-labeled gelatin and then disinfected using 70% ethanol. Residual free aldehydes were then quenched using complete media. The wells were protected from light for all remaining steps. Control or ADH1B-overexpressing SKOV3ip1 cells were plated on the wells at an even distribution. After 24 hours, the culture media was removed, and the cells were fixed with 3.7% formaldehyde in PBS. The wells were then washed with PBS and stained using a staining solution (FITC-phalloidin+DAPI+DPBS with 2% blocking serum + 0.25% Triton X-100). The wells were then incubated for 1 hour at room temperature. The wells were then washed with a florescent staining buffer followed by PBS. The chamber slides were then mounted using mounting media. Fluorescent microscopic imaging was performed using the following wavelengths: Cy3-Gelatin, Ex/Em = 550/570 nm; FITC-phalloidin, Ex/Em = 494/518 nm.

### Proteome profiler assay

For the proteome profiler assay, we used a proteasome profiler human protease array kit (cat. #ARY 021B, R&D Systems, Minneapolis, MN) following the manufacturer's instructions. Briefly, conditioned media from SKOV3ip1 cells ectopically expressing ADH1B and from control cells was collected. The protein concentration of the conditioned media was measured, and media containing 300 μg was used for the assay. Nitrocellulose membranes containing 35 protease capture antibodies were incubated using a blocking buffer. A protease detection antibody cocktail was incubated with the conditioned media for 1 hour at room temperature. After the incubation, the blocking buffer was removed, and the membranes were incubated overnight with the antibody-conditioned media mixture at 4° C. The membranes were then washed with a washing buffer and incubated with 1X streptavidin solution for 30 minutes at room temperature. The membranes were washed again, and an enhanced chemiluminescence detection kit (cat. #NEL104001EA, Pierce Biotechnology, Rockford, IL, USA) was used to visualize the signal. The positive signals were identified using the transparency overlay template provided in the kit.

### Adhesion assay

For the adhesion assay, we coated the wells of a 96-well plate with fibronectin (cat. # F1141, Sigma, St. Louis, MO). Mesothelial cells were then plated to form a monolayer on the layer of fibronectin. Control or ADH1B-overexpressing SKOV3ip1 cells were plated on the mesothelial cells. The cells were washed at 15, 30, 60, and 90 minutes. The attached cells were fixed with paraformaldehyde. The adherent cells were then counted using an Olympus CKX41 microscope and the GFP channel. Five random fields were used for cell counting.

### Tumor microenvironmental factors

To assess the effect of hypoxia on ADH1B expression, we incubated cancer cells at 37° C in either 1% O_2_ atmosphere or in regular conditions. To investigate the effect of stromal cells on ADH1B expression in cancer cells, we treated the cancer cells with conditioned media from stromal cells. For cell collection, the cells were first incubated in serum-free media for 24 hours. The media was then collected and centrifuged at 1200 RPM for 5 minutes to separate any dead cells. After treating the cancer cells with conditioned media, we collected the cells and measured ADH1B expression using quantitative real-time polymerase chain reaction (PCR).

### Quantitative real-time PCR

For quantitative real-time PCR, total RNA was extracted from the cancer cells using the Direct-Zol RNA extraction kit (Zymo Research, Irvine, CA, USA). RNA was then quantified using a NanoDrop spectrophotometer, and RNA quality was determined by checking 260 nm/280 nm ratios. RNA (1 μg per sample) was reverse-transcribed into cDNA using the Verso cDNA kit (Thermo Scientific, West Palm Beach, FL) according to the manufacturer's protocol.

Quantitative real-time PCR was performed on a 7500 PCR system (Applied Biosystems, Warrington, UK) using 1 μl of cDNA for each sample and 20 pmol of primer for the reaction. SYBR green (Applied Biosystems) was used to detect the products. All reactions were carried out with 20 μl of reaction mix and performed in triplicate. We used the following primers: for ADH1B, 5ʹAGGGTAGAGGAGGCTGAAGA3ʹ (forward), 5ʹACC TGCTTCACTCTGGGAAA3ʹ (reverse); for CD44, 5ʹAC TTGGCTTTCTGTCCTCCA3ʹ (forward), 5ʹ CCCAGAT GGAGAAAGCTCTG3ʹ (reverse); for CD47, 5ʹAGCA TGGAATGACGACAGTG3ʹ (forward), 5ʹGATGTGGC CCCTGGTAGC3ʹ (reverse); and for 18S, 5ʹCGCCGCTAG AGGTGAAATTC3ʹ (forward), 5ʹTTGGCAAATGCT TTCGCTC3ʹ (reverse). The following conditions were used for PCR: 50° C for 2 minutes, then 95° C for 15 minutes, and then 40 cycles at 95° C for 1 minute each. The reactions were analyzed using the 7500 Applied Biosystems PCR software (v.2.0.5). The cycle threshold values of the target genes were normalized to the cycle threshold values of 18S rRNA. The melt curves were checked to determine the specificity of the reactions.

### Copy number analysis

For the copy number analysis, we retrieved TCGA mRNA microarray data obtained with Agilent and Affymetrix platforms (Agilent 244K Custom Gene Expression G4502A-07 array, Affymetrix Human Genome U133A 2.0 Array, and Affymetrix Human Exon 1.0 ST Array) as well as RNASeqv2 level 3 data and clinical data from Broad GDAC Firehose (http://gdac.broadinstitute.org/). Putative copy numbers from GISTIC were retrieved from cBioPortal (http://www.cbioportal.org/). To assess the relationship between ADH1B expression and copy number, we first employed a Shapiro-Wilk test and verified that the data do not follow a normal distribution. We applied the non-parametric Kruskal-Wallis test and found no relationship between ADH1B and copy number. Data were visualized with a box-and-whisker plot, in which boxes represent the first (lower bound) and third (upper bound) quartiles and whiskers represent 1.5 times the interquartile range (log2[x]).

### Immunostaining

Paraffin-embedded, 5-μm sections of tumor tissues were subjected to immunostaining for detection of ADH1B. The formalin-fixed sections were deparaffinized by sequential washings with xylene, 100% ethanol, 95% ethanol, 80% ethanol, and PBS. A suitable antigen retrieval method was used. Endogenous peroxidase was blocked by incubating the slides with 3% hydrogen peroxide in PBS. The slides were then incubated with 4% fish gelatin to prevent nonspecific binding and then incubated with primary antibody (cat. #AV41787, Sigma) overnight at 4° C. The next day, the slides were washed with PBS and incubated with biotinylated antibody (4+biotinylated goat anti-rabbit immunoglobulin G; Biocare Medical, GR602H) for 20 minutes at room temperature. The slides were then washed with PBS and incubated with streptavidin HRP (4+ streptavidin HRP label, HP604H) for 20 minutes at room temperature. The slides were again washed with PBS and developed with 3,3ʹ-diaminobenzidine. The nuclei were stained with Gill's hematoxylin solution, and the slides were then mounted.

### Microarray and pathway analysis

Microarray was performed on Human HT-12 v4 Beadchip (Illumina) as per the protocol. The two groups analyzed were- control cells and cells ectopically expressing ADH1B (A2780 ip1). The mirVana RNA isolation labeling kit (Ambion) was used to extract total RNA from the cells. The purity and concentration of RNA were measured by a Nanodrop spectrophotometric measurement (Thermo Scientific). Five hundred nanograms of total RNA were used for the gene array. The microarray data were normalized using the quantile normalization method in the Linear Models for Microarray Data package in the R language environment. The expression level of each gene was then transformed into a log_2_ base. The pathway analysis was conducted using Ingenuity pathway analysis software.

### Zymography

We performed zymography on a gelatin-containing gel using conditioned media from control cells and from cells ectopically expressing ADH1B. Conditioned media from Siha cells was used as a positive control. To prepare the media for loading onto the gel, we diluted it using collagenase buffer and non-reducing sample buffer. The gel was run for 100 V at room temperature. After the electrophoresis, the gel was washed with water, incubated in renaturing buffer for 30 minutes, again washed with water, and then incubated with developing buffer for 30 minutes. The gel was then stained and visualized for the gelatin degradation.

### Statistical analysis

Statistical analyses were performed using Student's *t* test to compare the difference between control and treatment group. The test was two sided and error bars represent the standard error. *P* value less than 0.05 is considered to be statistically significant. Survival analysis and graphical representation was performed in R (version 3.2.5). The relationship between overall survival, and progression free survival was examined using a Cox proportional hazard model, using mRNA expression levels and clinical parameters (age, stage and grade) as covariates. A multivariate Cox proportional hazard model was fitted, including the clinical parameters and mRNA expression significant in the univariate analysis. The cohort is Tothill RW *et al*. [32] To visualize the data we first used the log-rank test to find the point (cut-off) with the most significant (lowest *p*-value) split in high, low ADH1B expression groups and then used the Kaplan-Meier method to generate plots for this cutoff (0.48 for overall survival and 0.53 for progression free survival). The number of patients at risk in the high, low mRNA groups at different time points are presented at the bottom of the graph.

## SUPPLEMENTARY MATERIALS FIGURES AND TABLES


