# Supplementary material for: ADH1B promotes mesothelial clearance and ovarian cancer infiltration
Source: Oncotarget. 2018 May 18;9(38):25115–26. doi: 10.18632/oncotarget.25344 (PMC5982754; doi:10.18632/oncotarget.25344)
Supplement: Supplementary file 1 [file oncotarget-09-25115-s001.pdf]

ADH1B promotes mesothelial clearance and ovarian cancer infiltration

SUPPLEMENTARY MATERIALS

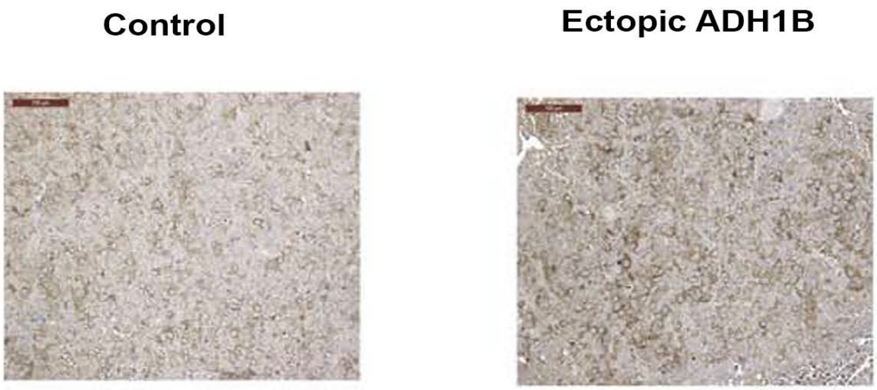

Supplementary Figure 1: ADH1B expression in tumor tissues from mice injected with ADH1B-overexpressing cells or control cells.

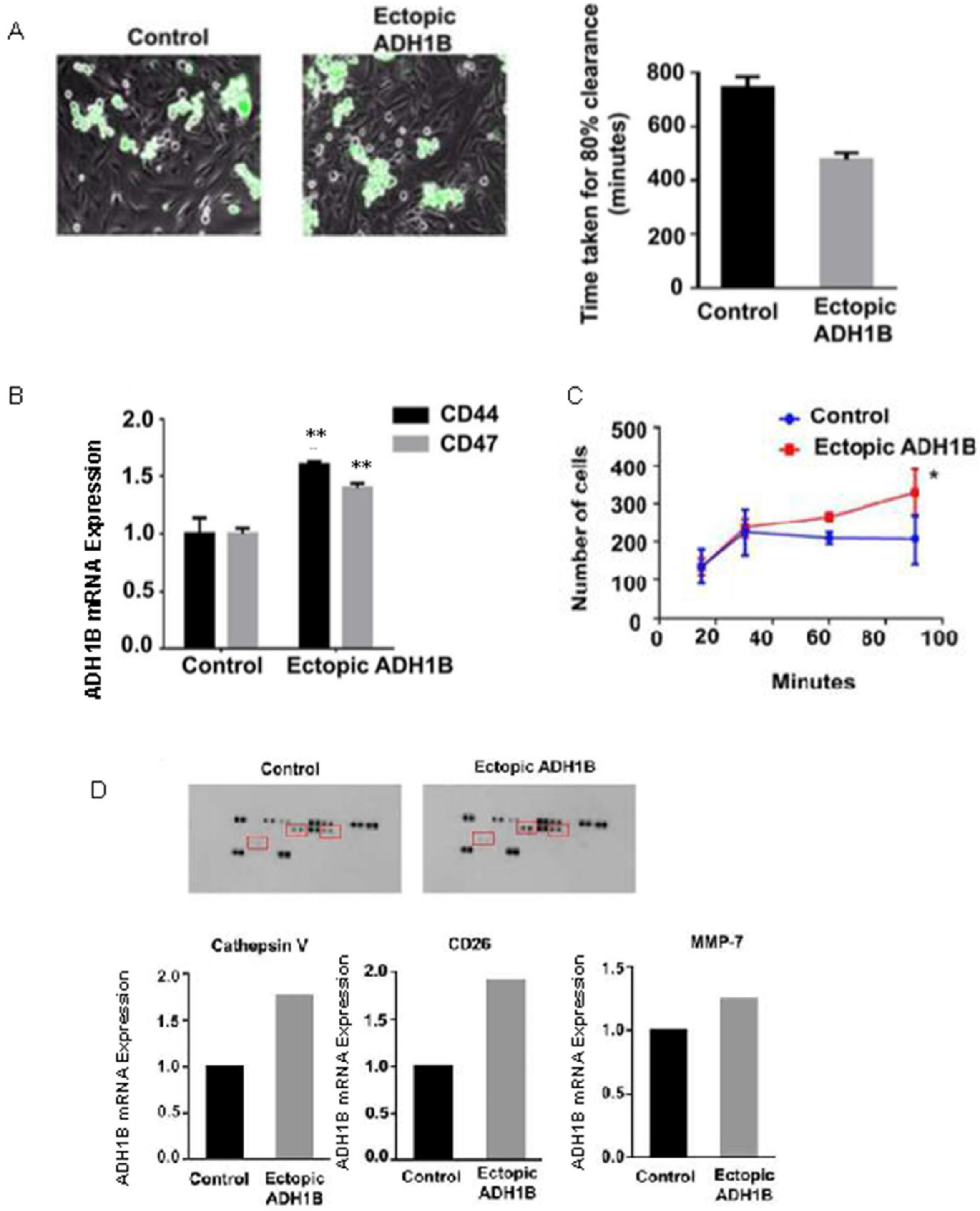

Supplementary Figure 2: (**A**) Representative images of live cell imaging conducted to visualize mesothelial clearance. Mesothelial cells were treated with conditioned media from cells with ectopic ADH1B expression or from control cells (SKOV3ip1). (**B**) ADH1B upregulation increased CD44 and CD47 expression. $^{**}p < 0.001$ is for CD44 and $p < 0.001$ CD47 expression in ectopic ADH1B cells vs. control. (**C**) ADH1B upregulation increased the number of SKOV3ip1 cells adhering to the mesothelial cell layer. $^{*}p < 0.05$. (**D**) Protease proteome profiler array blots show increases in cathepsin V, CD26, and MMP-7 in conditioned media from ADH1B-overexpressing cells upregulation. Data for all figures are represented as mean ± SEM.

A

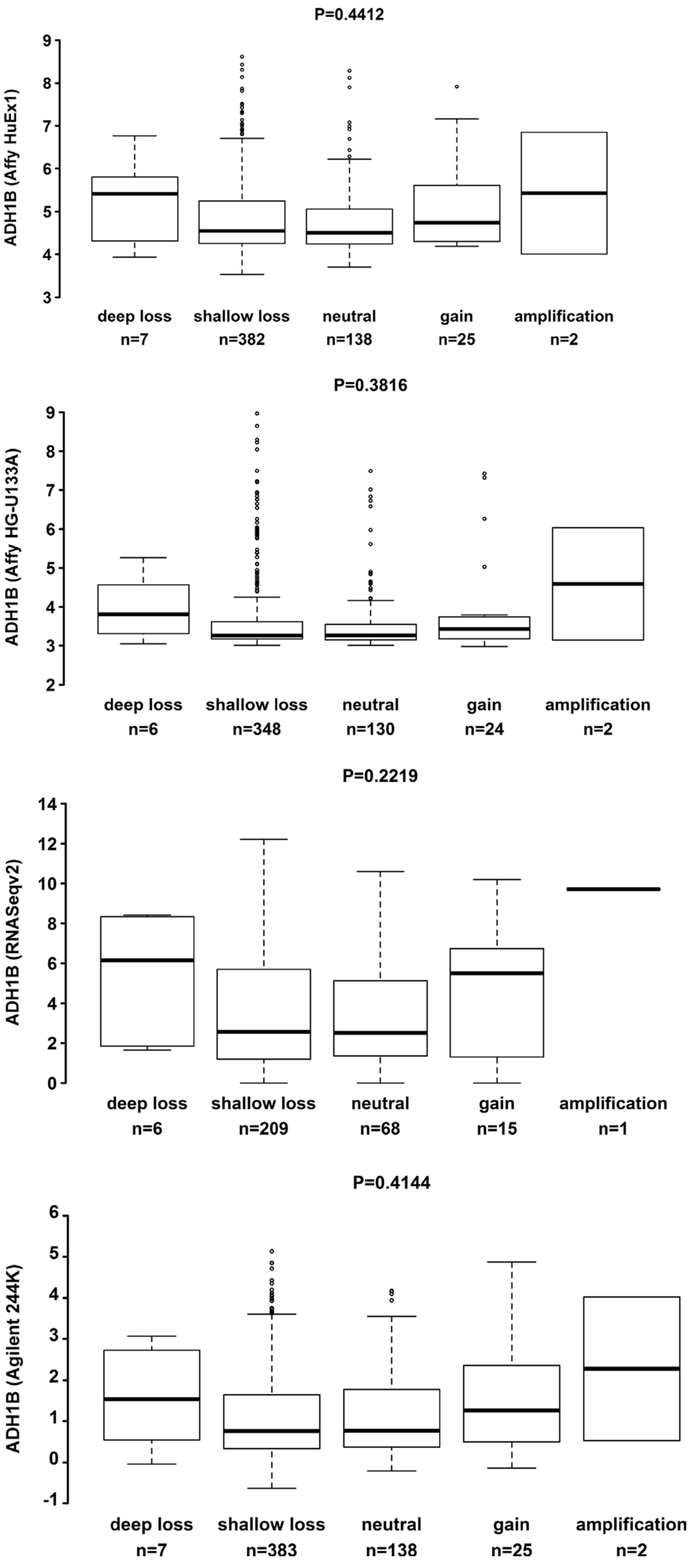

P=0.4412
ADH1B (Affy HuEx1)
deep loss n=7
shallow loss n=382
neutral n=138
gain n=25
amplification n=2
P=0.3816
ADH1B (Affy HG-U133A)
deep loss n=6
shallow loss n=348
neutral n=130
gain n=24
amplification n=2
P=0.2219
ADH1B (RNASeqv2)
deep loss n=6
shallow loss n=209
neutral n=68
gain n=15
amplification n=1
P=0.4144
ADH1B (Agilent 244K)
deep loss n=7
shallow loss n=383
neutral n=138
gain n=25
amplification n=2

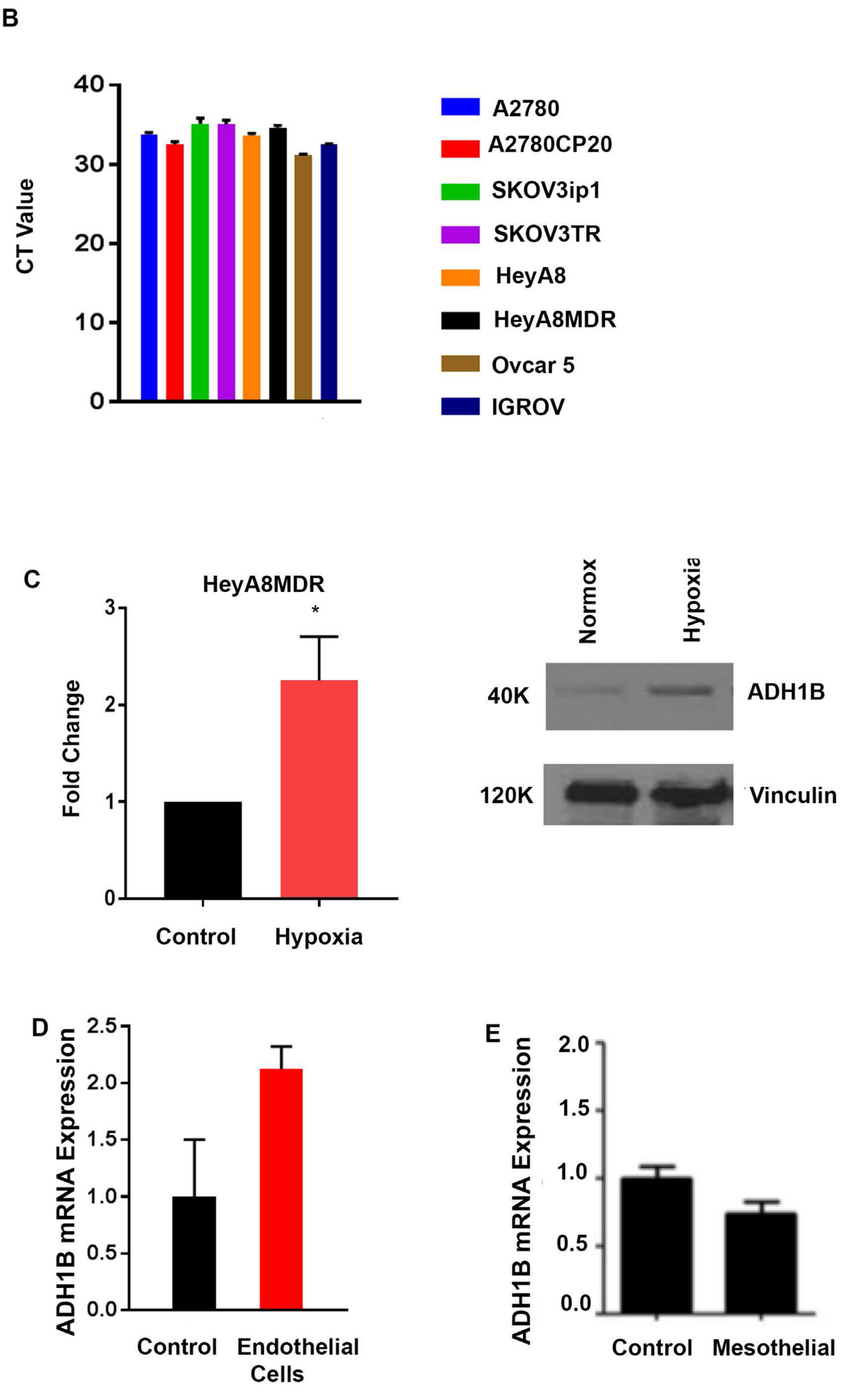

Supplementary Figure 3: (**A**) Correlation between copy number and mRNA expression of ADH1B across Affymetrix and Agilent platforms using TCGA ovarian cancer patient samples. (**B**) Expression of ADH1B across a panel of ovarian cancer cell lines. (**C**). Effect of hypoxia on the expression of ADH1B in HEYA8 MDR cancer cells. $^{*}p < 0.05$ is for ADH1B expression in hypoxic cells vs. normal conditions. ADH1B protein overexpression validated by western blot. (**D**) Effect of conditioned media from endothelial cells on ADH1B expression in HeyA8 MDR cells. $^{*}p < 0.05$ is for ADH1B expression in conditioned medial endothelial cells vs control. (**E**) Effect of conditioned media from mesothelial cells on ADH1B expression in HeyA8 MDR cells. Data for all figures are represented as mean ± SEM.
